# The research process and naivety–a student’s perspective

**DOI:** 10.15694/mep.2017.000175

**Published:** 2017-10-06

**Authors:** Arjuna Thakker

**Affiliations:** 1 University of Birmingham College of Medical and Dental Sciences

**Keywords:** Medical Student, Research

## Abstract

This article was migrated. The article was marked as recommended.

## Letter

Dear Editor,

When conducting research, medical students must take into account both the unpredictability of research work and the challenges posed by possible setbacks, especially when doing bench research. Experiments may not always go as planned, may have to be repeated or altered, and data collection can often be threatened by unanticipated variables.

Having completed research as part of an intercalated degree, I have come to appreciate these challenges as part of an entire research process ‘behind’ producing a journal article. For this reason, like others have also concluded, I encourage other students to intercalate. However, not been picked up in literature and what my own experience has suggested, is that despite whether students decide to intercalate or not, there is perhaps a bigger issue amongst students with regards to this research process, naivety.

A student’s first introduction into research usually occurs during pre-clinical teaching, where reading medical literature forms a key part of the curriculum, at any medical school. At Birmingham Medical School for example, second year students are required to complete a personal interest module (PIP), demonstrating an ability to understand experimental studies from scientific papers. For us students, this module serves as a first proper introduction into the field of scientific research. During this module however, we are only ever exposed to the end product of research, the journal article. Therefore, perceptions of research are being skewed, inclining only to think about the finished journal article, rather than understanding and arguably more importantly, appreciating the research process behind producing the journal article.

Approaches to counter balance this could involve integrating an understanding of the research process into the pre-clinical curriculum. Seminars could be given by both researchers and students who are actively involved in research, providing valuable insight to both the challenges they faced and the realities of undertaking research work. These seminars could be given as part of dedicated research modules, such as Birmingham’s PIP module, in order for students to develop a more rounded view of research, rather than that of just empirical results. Even better, part of this module could also involve allocating time to students for shadowing or even assisting assigned researchers in the labs. This would ensure that students become familiarized with the practicalities of bench research at an early stage in their training.

Both these approaches remain optimistic in nature. However, they would ensure that for students who eventually decide against intercalation, relevant awareness of the research process is still provided in their medical curriculum. On the other hand, for the intercalating student, early awareness of the research process would only serve to ease the transition (from medicine to research) that students make when intercalating. This is something in particular that I would have both appreciated and benefited from prior to my own intercalation.

The examples given above also conform to a trend of articles published on developing research skills in medical students. One specific example, an AMEE guide no.69 ( Laidlaw et al. 2012), encourages the integration of ‘research skills and associated attributes’ into the medical curriculum through methods such as those I have proposed above.

## Notes On Contributors

Arjuna Thakker is a current 3rd year medical student at the University of Birmingham. He recently completed a BSc in Anatomy and Human Biology at the University of Liverpool, where he undertook research work in the field of cellular and molecular physiology.
