# Supplementary material for: The research process and naivety–a student’s perspective
Source: MedEdPublish (2016). 2017 Oct 6;6:175. [Version 1] doi: 10.15694/mep.2017.000175 (PMC10885300; doi:10.15694/mep.2017.000175)
Supplement: Peer review history

# Reviewer report: Reviewer response for version 1

**DOI:** 10.21956/mep.19368.r28346

**Published:** 2018-09-29

**Authors:** Anthony David.M

**Affiliations:** 1 Soho

This review has been migrated. The reviewer awarded 4 stars out of 5 An interesting point view from an enduser. Research is being encouraged in medical students in the developing world. But there is some laxity from the majority of students as they are strategic learning and their learning is mainly assessment driven.But some initiatives such as short term research projects supervised by faculty are being taken up by interested students. These projects give a hands-on experience to the students on research work. The students are encouraged to publish their work also at a later stage.

Reviewer Expertise:

NA

No decision status is available

# Reviewer report: Reviewer response for version 1

**DOI:** 10.21956/mep.19368.r28347

**Published:** 2017-11-16

**Authors:** Susan Van Schalkwyk

**Affiliations:** 1 Stellenbosch University

This review has been migrated. The reviewer awarded 4 stars out of 5 This article offers an honest reflection on some of the challenges we really all experience the first time we are exposed to 'research' as an idea, a desired outcome and a scholarly activity. The final artefact (or outcome) of a research endeavour - typically the journal article - can indeed be regarded as 'false advertising', for the novice who must try to work back from what is presented in a neat, tight text. It can probably be quite daunting on the one hand, or encourage the sort of naivety that the author refers to here on the other. As he indicates, there is a currently body of literature that speaks directly to the value of integrating research into medical and related programmes quite early on. His recommendations in this regard are, therefore, useful.

Reviewer Expertise:

NA

No decision status is available

# Reviewer report: Reviewer response for version 1

**DOI:** 10.21956/mep.19368.r28345

**Published:** 2017-10-28

**Authors:** Paolo Colet

**Affiliations:** 1 Nazarbayev University

This review has been migrated. The reviewer awarded 5 stars out of 5 The author gives a new life into what can be a daunting topic by sharing his insights and enthusiasm in learning research. I agree with the author that learning and understanding research is to be involved in the process, rather than listening to it alone. Importantly, Thakker offers students a proactive way to take part and learn research for the duration of their study. Notably, the author’s reference to the AMEE guide no. 69 is rightfully included, as this would serve as an invitation to the readers, including educators to come about for an in-depth discussion about the topic. Indeed, this is an excellent piece of article.

Reviewer Expertise:

NA

No decision status is available

# Reviewer report: Reviewer response for version 1

**DOI:** 10.21956/mep.19368.r28344

**Published:** 2017-10-26

**Authors:** P Ravi Shankar

**Affiliations:** 1 American International Medical University

This review has been migrated. The reviewer awarded 4 stars out of 5 This is an interesting article by a medical student about the research process. The author provides the perspective of a student who has completed an intercalated degree. Being involved in research has a number of advantages for students which have been listed in the literature. Medical students in most medical schools learn to critically analyze and interpret different types of publications and to base their decisions on evidence. An outline or an overview of the research process is often provided during the course but students are not introduced to the complexities and the unpredictability of research. Student participation in research projects could be one method of providing this perspective. In many medical schools in the Caribbean facilities for ‘bench’ research are limited and the challenges peculiar to this type of research may not be appreciated by students. I agree with Dr Gibbs, the other reviewer that obtaining ideas and insights from students is important and can strengthen teaching-learning and guide curricular development.

Reviewer Expertise:

NA

No decision status is available

# Reviewer report: Reviewer response for version 1

**DOI:** 10.21956/mep.19368.r28343

**Published:** 2017-10-10

**Authors:** Trevor Gibbs

**Affiliations:** 1 AMEE

This review has been migrated. The reviewer awarded 4 stars out of 5 It is always good to read the student perspective on curricula structure and development. I thought that the words and feelings that came out of this letter reflected an important issue amongst many students and many medical schools. Setting an activity within a curriculum in the hope of addressing a specific learning objective can be difficult, but getting it right is more important and gaining insight and ideas from students is an excellent way to progress.

Reviewer Expertise:

NA

No decision status is available
